# The emerging role of oxylipins in thrombosis and diabetes

**DOI:** 10.3389/fphar.2013.00176

**Published:** 2014-01-07

**Authors:** Benjamin E. Tourdot, Intekhab Ahmed, Michael Holinstat

**Affiliations:** ^1^Cardeza Foundation for Hematologic Research, Department of Medicine, Thomas Jefferson UniversityPhiladelphia PA, USA; ^2^Division of Endocrinology, Diabetes and Metabolic Diseases, Department of Medicine, Thomas Jefferson UniversityPhiladelphia PA, USA

**Keywords:** cardiovascular disease, oxylipins, diabetes, thrombosis, hemostasis, polyunsaturated fatty acids, 12-lipoxygenase, lipoxygenase

## Abstract

The prevalence of cardiovascular disease (CVD), the leading cause of death in the US, is predicted to increase due to the shift in age of the general population and increase in CVD risk factors such as obesity and diabetes. New therapies are required to decrease the prevalence of CVD risk factors (obesity and diabetes) as well as reduce atherothrombosis, the major cause of CVD related mortality. Oxylipins, bioactive metabolites derived from the oxygenation of polyunsaturated fatty acids, play a role in the progression of CVD risk factors and thrombosis. Aspirin, a cyclooxygenase-1 inhibitor, decreases atherothrombotic associated mortality by 25%. These potent effects of aspirin have shown the utility of modulating oxylipin signaling pathways to decrease CVD mortality. The role of many oxylipins in the progression of CVD, however, is still uncertain or controversial. An increased understanding of the role oxylipins play in CVD risk factors and thrombosis could lead to new therapies to decrease the prevalence of CVD and its associated mortality.

In the US, cardiovascular disease (CVD) is responsible for 25% of all deaths making it the leading cause of mortality (Murphy et al., 2013). CVD is a group of diseases that affects the heart or blood vessels including heart failure, hypertension, coronary artery disease, and cerebral vascular disease. Thrombotic complications of coronary artery and cerebral vascular disease are largely responsible for the high level of mortality associated with CVD (Jackson, 2011; Roger et al., 2011) and current anti-platelet therapy has successfully reduced CVD associated mortality by approximately 25% (Jackson, 2011; Roger et al., 2011). While those results are encouraging there is growing concern over the increase in the population with CVD risk factors such as obesity and diabetes. Diabetic patients are disproportionately affected by CVD which is responsible for 65% of patient mortality (Lincoff, 2003). Over 7.7% of the US population is diagnosed with diabetes and another 29% is diagnosed as pre-diabetic (Lloyd-Jones et al., 2010). Hence, newer therapies are required to decrease CVD risk factors for diabetics. An underutilized therapeutic approach to treating these diseases is through modulation of oxylipin signaling. Oxylipins are bioactive lipids generated by the oxidation of polyunsaturated fatty acids (PUFAs). Recent research has designated a role for oxylipins in both CVD as well as diabetes. While the cyclooxygenase (COX)-derived oxylipins have been the focus of many reviews, the goal of this review is to shed light on the emerging roles of 12-lipoxygenase (12-LOX) in the pathologies of thrombosis and hemostasis as well as progression of diabetes.

## OXYLIPIN BIOLOGY

**FIGURE 1:**
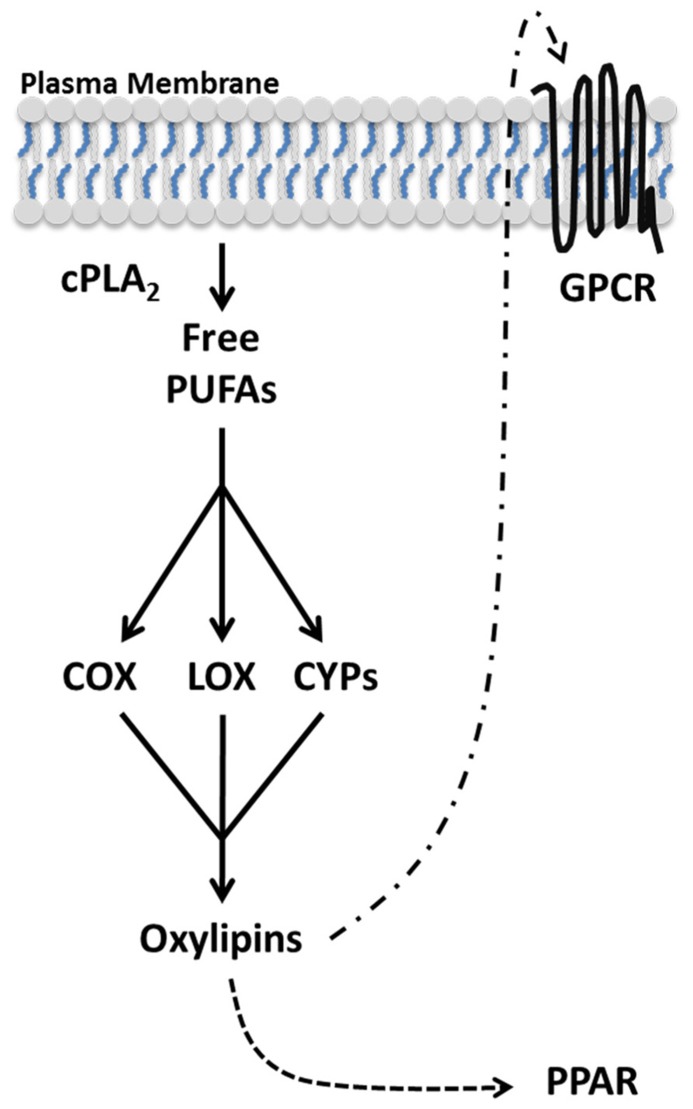
** Oxylipin biosynthesis and signaling**. Oxylipins are synthesized *de novo* from polyunsaturated fatty acids (PUFAs) in an activation dependent manner. Upon cellular activation, cPLA_2_ hydrolyzes PUFAs from the lipid membrane generating free PUFAs. Oxygenases (COX, LOX, and CYP) metabolize free PUFAs into distinct oxylipins. Oxylipins can diffuse through the plasma membrane and bind GPCRs in the local environment. Additionally, select oxylipins can activate the transcription factor PPAR.

Oxylipins follow a general biosynthesis and signaling scheme (**Figure 1**). Due to the potency and short half-life of most oxylipins, they are not stored but synthesized *de novo* in a tightly regulated manner (Funk, 2001). In order to restrict aberrant oxylipin production, the level of free PUFAs are kept low with the preponderance of PUFAs actively sequestered to membrane-bound glycerophospholipids by the action of acyltransferases and transacylases (Perez-Chacon et al., 2009). Upon cellular activation, PUFAs in the sn-2 position of membrane glycerophospholipids are liberated by cytoplasmic phospholipase A_2_ (PLA_2_; Ghosh et al., 2006). Free PUFAs are oxygenated by three families of enzymes COX, lipoxygenase (LOX), and cytochrome P450 (CYP) into distinct classes of oxylipins (Massey and Nicolaou, 2013). Subsequently, oxylipins can activate peroxisome proliferator-activated receptors (PPARs), ligand activated transcription factors, or diffuse through the plasma membrane and signal through G protein-coupled receptors (GPCRs) in a paracrine or autocrine manner (Shearer and Newman, 2009; Wahli and Michalik, 2012). The type of oxylipins produced depends predominately on the PUFA being oxidized and the oxygenase metabolizing the PUFA.

The oxygenation of different PUFAs gives rise to distinct oxylipins that vary in length and double bond configuration. These parameters determine the oxylipins three-dimensional spatial configuration and receptor specificity (Mozaffarian and Wu, 2012). The main oxylipin PUFA precursors [dihomo-gamma-linolenic acid (DGLA), arachidonic acid (AA), eicosapentaenoic acid (EPA), and docosahexaenoic acid (DHA)] can be obtained directly from the diet or from the elongation and desaturation of linolenic (omega-3, 18:3) and linoleic acid (LA; omega-6, 18:2) and alpha-linolenic acid (ALA), respectively (Massey and Nicolaou, 2013).

The linoleic acid metabolism pathway is responsible for the production of two main oxylipin precursors, DGLA and AA (Lagarde et al., 2013). The first step in this pathway is the addition of a double bond to LA by Δ^6^ desaturase which produces gamma-linolenic acid (GLA, 18:3). An elongase then facilitates the addition of two-carbons to GLA forming DGLA (20:3). DGLA is a substrate for oxygenases but can be further converted to AA (20:4), the most abundant oxylipin precursor, through the addition of a double bond by Δ^5^ desaturase (Lagarde et al., 2013). The activity of Δ^5^ desaturase in human platelets, monocytes and neutrophils is limited; therefore supplementation with either GLA or DGLA does not increase AA levels in these cells (de Bravo et al., 1985; Pullman-Mooar et al., 1990; Barre and Holub, 1992; Chilton et al., 1996).

**FIGURE 2:**
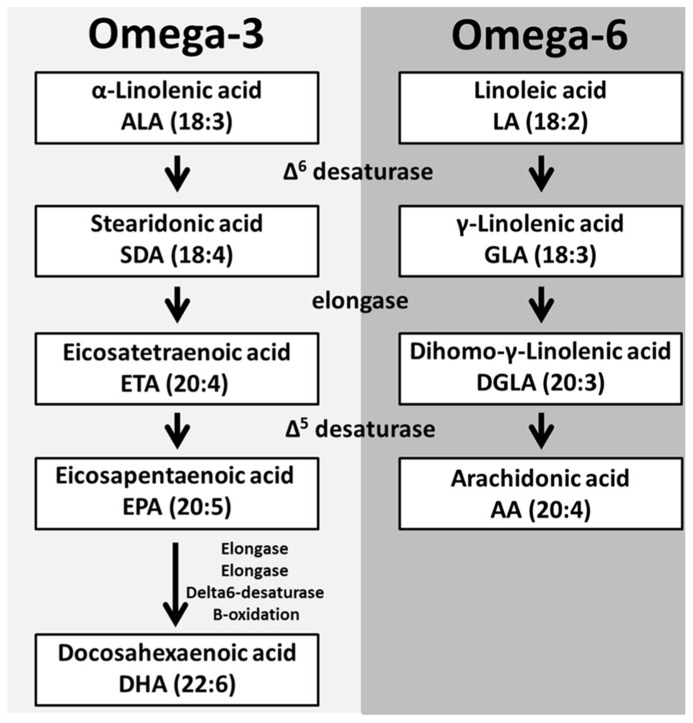
** Polyunsaturated fatty acid biosynthesis.** The essential fatty acids α-linolenic acid (ALA) and linoleic acid (LA) are metabolized to produce PUFAs. The initial step is the addition of a double bond to both ALA and LA to form the respective desaturated products. These desaturated metabolites are elongated and another desaturase can add a double bond to these elongated products to produce EPA and AA, respectively. EPA through a series of enzymes is converted into DHA.

**FIGURE 3:**
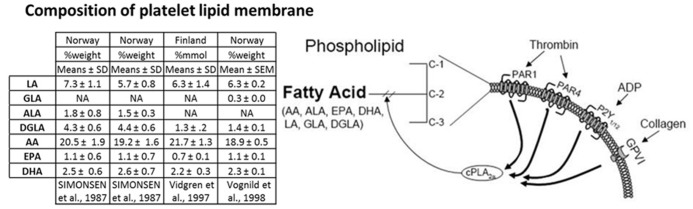
** PUFA platelet membrane composition and agonist induced PUFA membrane liberation.** The table displays the amount of PUFAs contained in the platelet membrane of different European populations from three studies as %weight or %mmol compare to total fatty acids. Columns 1 and 2 of the table represent data from the same study comparing the platelet composition of individuals from either inland (column 1) or coastal communities (column 2) in northern Norway. Liberation of PUFAs from the platelet membrane is achieved in an agonist dependent manner. Platelet stimulation through a myriad of agonists including thrombin, ADP, and collagen cause the translocation of cPLA_2__α_ to the plasma membrane where it cleaves PUFAs from the plasma membrane to generate free PUFAs, which can be metabolized to oxylipins.

The other essential PUFA, ALA (18:3) is converted into the oxylipin precursors EPA and DHA in a similar manner to the metabolism of LA (Lagarde et al., 2013; **Figure 2**). These PUFAs are esterified into the sn-2 position of glycerophospholipids and stored in the lipid membrane providing a potentially high level of substrate available for *de novo* production of bioactive lipids (Lagarde et al., 2013; **Figure 3**).

Active cPLA_2_ is required to hydrolyze PUFAs from the plasma membrane. The importance of cPLA_2_ to liberate PUFAs has been highlighted by a patient who is functionally deficient in cPLA_2__α_ (Reed et al., 2011). Oxylipin production was reduced by 95% in the cPLA_2__α_ deficient patient compared to healthy controls following blood coagulation (Adler et al., 2008). The mechanism by which free PUFAs traffic to oxygenases is not fully elucidated. Recent data in platelets suggests that there are two different pools of AA that can be selectively utilized by COX-1 and 12(S)-LOX (Holinstat et al., 2011). Further studies are required to determine how free PUFA is relegated amongst the various oxygenase isozymes expressed in different cell types.

Cyclooxygenase, one of the major oxygenases, converts PUFAs into prostanoids, a subgroup of oxylipins. Prostanoids contain one or more double bonds and a characteristic five-membrane ring structure from carbons 8–12. Ring structures are designated by different letters from A to K, recently reviewed in detail (Buczynski et al., 2009). COX converts DGLA, AA, and EPA into series one prostanoids (PGD_1_, and PGE_1_), series two prostanoids (PGD_2_, PGE_2,_ PGF_2_, PGI_2_, and TxA_2_) and series three prostanoids (PGE_3_, PGD_3_, and TxA_3_), respectively (Zivkovic et al., 2012). Prostanoids can diffuse through the plasma membrane and bind to GPCRs on the surface of cells in a paracrine or autocrine manner. The number of double bonds and type of ring structure in a prostanoid helps establish its prostanoid receptor specificity. The prostanoid receptors are divided into five classes [Prostanoid D receptor (DP), Prostanoid E receptor (EP), prostacyclin receptor (IP), thromboxane receptor (TP), and Prostanoid F receptor (FP)] characterized by their most potent biological ligand, however, there is ligand cross reactivity with these receptors. For more in depth review on prostanoids and their receptors, please see Bos et al. (2004).

Lipoxygenases are dioxygenases that catalyze the hydroperoxidation (-OOH) of PUFAs to form oxylipins such as leukotrienes, lipoxins, and hydroxyeicosatetraenoic acids (HETEs; Strassburg et al., 2012). Humans express six AA lipoxygenase (ALOX) genes that can be categorized into four groups of LOX enzymes (3-LOX, 5-LOX, 12-LOX, and 15-LOX) according to the specific carbon of AA they oxidize. LOX isozymes are further characterized by tissue expression and stereospecificity (R or S; Horn et al., 2013). This nomenclature, however, can be misleading as LOX isozymes can oxidize other PUFAs as well as oxidize these PUFAs at carbons unique from those oxidized on AA. The LOX-derived oxylipins including HETE and leukotrienes are able to freely pass through the plasma membrane and signal through GPCRs.

Cytochrome P450 enzymes are a diverse array of membrane bound, hemeproteins named for their unique absorbance peak at 450 nm when reduced and bound by carbon monoxide. The 57 CYPs expressed in humans are broken into 18 families and 43 subfamilies based on conserved amino acid identity. CYP nomenclature uses a number to identify the family, a letter to categorize the subfamily and a subsequent number to identify the isozyme, for example, CYP2C19 (McKinnon et al., 2008). CYP family members are best known for their role in xenobiotic metabolism, but can also metabolize endogenous molecules such as PUFAs to produce oxylipins. For example, CYP isozymes with hydrolase activity (CYP4A and CYP4F) generate HETEs while CYPs with epoxygenase activity (CYP2C and CYP2J) generate epoxyeicosatrienoic acid (EETs) that can be metabolized to dihydroxyeicosatrienoic acids (DHETs) by soluble epoxide hydrolase (sEH; Fleming, 2001; Panigrahy et al., 2010). Like other oxylipins, HETEs, EETs, and DHETs diffuse through the plasma membrane and bind to GPCRs on the surface of cells as well.

Oxylipins have a myriad of functions that are still being elucidated. Aberrant oxylipin signaling has been shown to lead to a number of pathologies important to CVD including hyperlipidemia, hypertension, thrombosis, and hemostasis (Gleim et al., 2012). While oxylipins play an important role in a number of physiological and pathophysiological conditions, this review will be limited to oxylipin regulation of hemostasis, thrombosis, and diabetes.

## OXYLIPINS IN HEMOSTASIS AND THROMBOSIS

The study of oxylipins and hemostasis began with an anecdotal observation by Lawrence Craven in the 1940s that tonsillectomy patients given aspirin gum as an analgesic had an increased risk of hemorrhage (Miner and Hoffhines, 2007). Spurred by this observation he began prescribing patients with high risk of a heart attack low doses of aspirin, he observed no incidence of CVD related mortality in the approximately 8000 people he treated with aspirin therapy (Miner and Hoffhines, 2007). Unfortunately, his studies lacked the rigor required to prove that aspirin did in fact lower the incidence of myocardial infarction. In the late 1960s, aspirin was shown to reduce platelet aggregation by irreversibly inhibiting COX, thus inhibiting the production a potent prothrombotic oxylipin, thromboxane A_2_ (Miner and Hoffhines, 2007). It was not until 1989 that the Physicians’ Health Study provided the scientific rigor to conclude that aspirin does reduce the rate of myocardial infarction (Hennekens, 1989). Blocking secondary mediators of platelet activation such as TxA_2_ raise the threshold of platelet activation, but not completely ablate platelet function. Other than COX metabolites, the functions of only a few oxylipins have been characterized in platelets and those that have been characterized still remain controversial (Lagarde et al., 2010; Ikei et al., 2012). The roles that LOX and CYPs play in regulating platelet function is less defined but both enzymes have been shown to signal in other cell types and therefore warrant further examination (Brash, 1985; Panigrahy et al.,2010).

Cyclooxygenase derived oxylipins are the most well characterized oxylipins in platelets. The activation status of platelets are regulated by the integration of pro-thrombotic signals by TxA_2_ and anti-thrombotic signals through PGI_2_ (Smyth et al., 2009). Platelets are producers of prostanoids (TxA_2_, PGE_2_, and PGD_2_) as well as the targets of prostanoids produced by other vascular cells such as endothelial cells and leukocytes (PGI_2_ and PGE_1_; Marcus, 1978). One of the primary effects of thromboxane is to potentiate platelet aggregation initiated by other agonists through a positive feedback mechanism. TxA_2_ signals through the thromboxane (TPα ) receptor expressed on the surface of platelets and increases intracellular calcium (Li et al., 2010). The primary anti-thrombotic prostanoid identified thus far, PGI_2_, is predominately synthesized by COX-2 in endothelial cells (Smyth et al., 2009). PGI_2_, as well as the other anti-thrombotic prostanoids PGD_2_, and PGE_1_, bind to their respective GPCRs on the surface of the platelet initialing an inhibitory signal (Marcus, 1978; Smyth et al., 2009; Iyú et al., 2011). The role of PGE_2_ is controversial with previous work determining it had pro- and anti-thrombotic functions. Consistent with these findings a recent study showed PGE_2_ can bind either a pro-thrombotic (EP_3_) or anti-thrombotic (EP_4_) receptor on the surface of platelets (Iyú et al., 2011). Elucidating the conditions under which PGE_2_ is anti-thrombotic could be therapeutically beneficial.

The effectiveness of low dose aspirin at reducing myocardial infarctions is believed to be due to its ability to inhibit the production of TxA_2_ from platelets, with only minimal disruption of PGI_2_ from endothelial cells. Consistent with this data two reports have found that non-steroidal anti-inflammatory drugs (NSAID), COX-1 and COX-2 inhibitors, use decreases the beneficial effects of aspirin (Kurth et al., 2003; MacDonald and Wei, 2003). While still controversial, the increased risk in heart attacks associated with specific COX-2 inhibitors (celecoxib and rofecoxib) is hypothesized to be due to a disruption in the balance of TxA_2_ and PGI_2_ creating a pro-thrombotic environment (Salinas et al., 2007; Cannon and Cannon, 2012; Yeung and Holinstat, 2012). Rather than ablate all prostanoid signaling it appears a more potent therapy is to manipulate the balance of pro and anti-thrombotic prostanoids. That lesson may be useful when studying other oxylipins signaling systems in the platelet such as LOX and CYP.

**FIGURE 4:**
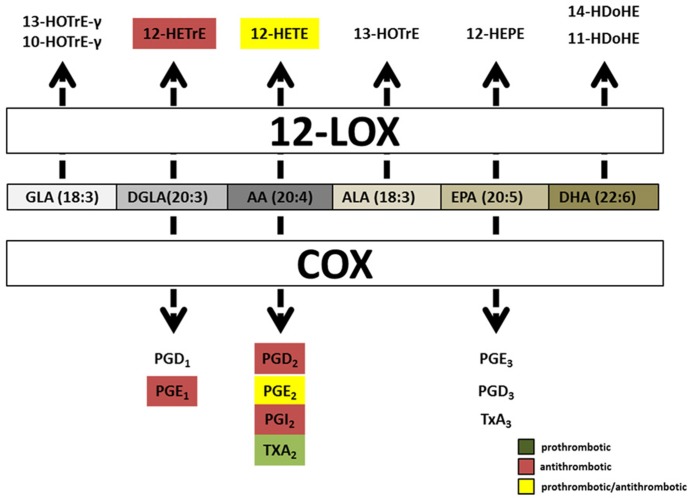
** Hemostatically active oxylipins derived from COX and platelet 12(S)-LOX.**Common oxylipin precursors (GLA, DGLA, AA, ALA, EPA, and DHA) are all substrates for platelet 12(S)-LOX while COX produces oxylipins predominately from EPA, DGLA, and AA. Both COX and platelet 12(S)-LOX generate oxylipins that have either prothrombotic (green box) or anti-thrombotic (red box) signaling properties. Oxylipins designated with yellow boxes have been reported as pro and anti-thrombotic in the literature.

The first LOX, platelet 12(S)-LOX, was discovered in humans in 1974, yet the role LOX enzymes play in hemostasis and thrombosis remains controversial (Hamberg and Samuelsson, 1974). 12(S)-LOX is predominantly expressed in platelets and their precursors, the megakaryocyte. It’s constitutively active in resting platelets with the majority of the protein located in the cytosol, and translocates to the lipid membrane during platelet activation in a Ca^2^^+^-dependent manner (Ozeki et al., 1999). 12(S)-LOX can oxygenate AA, DGLA, EPA, and ALA to produce single metabolites 12(S)-HPETE, 12(S)-HPETrE, 12(S)-HPEPE, and 13(S)-HPOTrE, respectively. However, DHA and GLA are 12-LOX substrates that produce two metabolites. The two DHA-derived metabolites can be divided into a major product, 14-HDoHE (66%), and a minor, 11-HDoHE (33%). GLA is also processed by 12-LOX into two unequally produced products, 10-HOTrE-γ (55%) and 13-HOTrE-γ (44%; Ikei et al., 2012; **Figure 4**). Two other PUFAs, EDA or LA, were incubated with 12-LOX but no oxygenated products were produced.

Two oxylipins derived from platelet 12(S)-LOX have been shown to have signaling properties in platelets, 12-HETE and 12-HETrE. 12(S)-HETE contains an extra carbon double bond between carbon 5 and 6 relative to 12(S)-HETrE, a result of the double bond configuration in their precursors AA and DGLA, respectively (Lagarde et al., 2010; Ikei et al., 2012). Platelet 12(S)-LOX processes AA and DGLA at the same rate, however, since AA is more abundant, 12-HpETE is found to be the predominate metabolite produced (**Figure 4**). 12-HpETE is immediately reduced to 12-HETE by glutathione reductase in platelets. In response to thrombin or collagen stimulation human platelets can produce an abundant amount of 12-(S)-HETE (40–60 ng/4 × 10^7^ platelets; Holinstat et al., 2011; Hammond and O’Donnell, 2012). 12-(S)-HETE by itself does not cause platelet aggregation rather it is believed to function in a positive feedback loop (Setty et al., 1992). The exogenous addition of 12-(S)-HETE to washed platelets has produced inconsistent results on platelet aggregation. Previous studies have reported that 12-(S)-HETE potentiates collagen, adenosine diphosphate (ADP) and protease-activated receptor (PAR) signaling, while other studies have shown that 12-(S)-HETE inhibits platelet aggregation in response to AA (Chang et al., 1985; Sekiya et al., 1990, 1991; Setty et al., 1992; Johnson et al., 1998; Katoh et al., 1998; Lagarde et al., 2010; Yeung and Holinstat, 2011). These differences could be due to variations in platelet preparation, species differences or amounts of agonist and 12-(S)-HETE used. Disagreement on the role of 12-(S)-HETE on platelet aggregation will persist until a definitive signaling pathway in platelets is determined. The identification of GPR31 as a high affinity GPCR for 12-(S)-HETE has demonstrated that like leukotrienes, 5-LOX derived oxylipins, 12-(S)-HETE can also signal via GPCRs (Murphy and Gijon, 2007; Guo et al., 2011). However, it remains to be determined if GPR31 is expressed and functional in platelets.

12(S)-HETrE, the other active platelet 12-(S)-LOX oxylipin, was just recently identified in platelets. As previously stated, 12(S)-HETrE is produced by the metabolism of DGLA by platelet 12-(S)-LOX. The exogenous addition of 12(S)-HETrE to washed platelets inhibited PAR-1, ADP, and collagen mediated platelet aggregation. DGLA and 12(S)-HPETrE, also inhibited clot retraction (Ikei et al., 2012). The anti-thrombotic effects of DGLA have been known for over 30 years, however, the mechanism by which DGLA regulates thrombosis remains unknown. (Kernoff et al., 1977) This data suggests that platelet 12-(S)-LOX metabolism of DGLA to 12(S)-HETrE is responsible for DGLAs anti-thrombotic effects. However, further work is required to confirm the role platelet 12-(S)-LOX has in the anti-thrombotic effects of DGLA.

Human disease conditions that result in decreased 12-LOX expression support a role for 12-LOX in thrombosis and hemostasis. Patients with myeloproliferative disorders that have a decrease in 12-LOX expression have an increase in bleeding and a reduction in thrombotic complications compared to myeloproliferative patients with normal 12-LOX levels (Schafer, 1982; Okuma et al., 1989; Matsuda et al., 1993). Additionally, patients deficient in the transcription factor, RUNX1 have altered 12-LOX expression and exhibit a bleeding diathesis (Kaur et al., 2010). While these results are consistent with 12-LOX playing a role as a regulator of hemostasis they are complicated by the fact that these patients may be deficient in other proteins, and the sample size was small.

The challenges of inhibiting 12-LOX specifically have been the topic of two recent reviews (Skrzypczak-Jankun et al., 2007; Yeung and Holinstat, 2011). While the specificity of previous so-called 12-LOX inhibitors (such as baicalein) have come into question (Yeung et al., 2012), newer highly selective inhibitors have been developed (NCTT-956), which confirm the role of 12-LOX in potentiating platelet activation (Yeung et al., 2012).

Evidence supporting a role for CYPs in regulation of oxylipins in platelets is lacking. Besides thromboxane synthase, CYP5A1, a well-known platelet CYP, the role CYPs play in platelet activation remains relatively unknown. The cyp-oxylipins EET and 20-HETE have been identified in platelets for years, but there was no evidence that the enzymes existed in platelets until recently (Zhu et al., 1995). One group has demonstrated CYP-derived oxylipins and CYP protein expression in platelets and their megakaryocyte precursors. The group was able to identify the mRNA and protein of three CYPs (CYP1A1, 2U1, and 2J2) in human megakaryocyte [megakaryoblastic leukemia (DAMI) cells]. Additionally, DAMI cells were able to produce 15 oxylipins, 5-,8-,9-,11-,12-,15-, 20-HETE, 11,12-EET, 14,15-EET, 5,6-DHET, 11,12-DHET, 14,15-DHET, in the presence of exogenous AA. These results were further demonstrated by the addition of a CYP inhibitor, SKF-525A that significantly reduced the production of EET, DHET, and HETE products (Jarrar et al., 2013b). Consistent with the study in DAMI cells, the same group was able to show that the addition of exogenous AA to washed platelets produced 17 metabolites which included the cyp-oxylipins EETs (8,9-, 11,12-, and 14,15-EET) and 20-HETE (Jarrar et al., 2013a). Consistent with DAMI cells mRNA and protein expression, CYP1A1, 2U1, and 2J2 were detected in platelets. On the other hand, both the mRNA and protein of two additional CYPs, 4A11 and 4F2, were detected in platelets, but were not found in the DAMI cell line. While the role of CYP-derived oxylipins in platelets is unknown, based on their signaling properties in other cells, it is hypothesized that they may have a signaling role in platelets as well. The EET receptor remains to be identified, however, evidence suggests that EETs can signal through GPCRs (Thomson et al., 2012). Follow-up studies are required to determine whether platelets express cyp-oxylipin receptors and if so what role they play in the regulation of platelet aggregation.

## OXYLIPINS AND DIABETES

The alarming increase in diabetes (type 1 and 2) and obesity has created an escalating global societal concern (Turner et al., 2013). Substantial evidence indicates that obesity induces a state of chronic low-grade inflammation resulting in insulin resistance (Barton, 2008). This obesity-induced inflammation exerts its detrimental effects on multiple cell types in the body including the insulin producing beta-cells (Asrih and Jornayvaz, 2013). During the last two decades, considerable effort has been made to elucidate the molecular factors responsible for obesity-induced inflammation. As a result, it is now well established that obesity-induced inflammation involves the similar set of molecules/signaling pathways to those involved in a classical inflammation including 12-LOX derived oxylipins. 12-LOX and its oxylipins are now known to play an important role in both adipogenesis as well as the destruction of beta-cells, both key pathologies in diabetes.

Studies of adipogenesis have revealed its dependence on an exogenous supply of free PUFAs and LOX-derived oxylipins to facilitate activation of PPAR (Madsen et al., 2003) in early adipocyte differentiation (Barak et al., 1999), which is evidenced by treatment of 3T3-L1 pre-adipocytes with either non-specific or specific 12-LOX inhibitors (Yu et al., 1995). In addition, this role appears to be specific to the epidermal-derived 12-LOX, as the platelet- and leukocyte-derived 12-LOXs are expressed at very low levels in the pre-adipocytes and early differentiated adipocytes. Additionally, no adipogenic defects were observed in leukocyte-12-LOX or platelet-12-LOX deficient mice (Kozak et al., 2002; Hallenborg et al., 2010). However, Leukocyte-12-LOX (12/15-LOX) appears to be a significant player in modulating adipocyte function *in vivo* in diet-induced mouse models of obesity. In a comparative study of 12/15-LOX knockout mice with C57BL6/J mice fed either a standard chow or high-fat “Western” type diet revealed that 12/15-LOX is the primary enzyme generating the 12(S)-HETE products under obese conditions (Nunemaker et al., 2008). The increased 12/15-LOX activity coincided with increased inflammation both systemically and in epididymal adipose tissue. Fewer incidences of macrophage infiltration and activation were observed in the epididymal adipose fat pads from 12/15-LOX knockout mice when fed the Western diet. Moreover, 12/15-LOX knockout mice also showed protection from developing insulin resistance and maintained normal adiponectin, an anti-inflammatory adipose-derived cytokine (adipokine; Sears et al., 2009). All this data indicates that 12/15-LOX activation under diet-induced obese conditions plays a significant role in mediating inflammation via ensuing adipocyte dysfunction. Chakrabarti et al. (2009) performed a detailed evaluation of the role of 12/15-LOX-derived products in adipocytes. In their experiments, direct addition of 12(S)-HETE and 12(S)-HPETE to differentiated 3T3-L1 adipocytes resulted in increased inflammatory cytokines, tumor necrosis factor-alpha (TNF-α), monocyte chemotactic protein-1 (MCP-1), interleukin (IL)-6, and IL-12p40, and a decreased expression of adiponectin. In addition, these products induced insulin resistance as measured by a decrease in insulin-mediated activation of key insulin-signaling proteins, such as Akt and insulin receptor substrate-1. Furthermore, a free fatty acid component of high-fat diets, palmitic acid was able to induce 12/15-LOX expression in 3T3-L1 adipocytes, demonstrating that products of 12/15-LOX pathway can directly impair adipocyte function in a fatty acid surplus environment.

Upregulated 12-LOX activity or expression have also been implicated in the functional loss of insulin secretion or production in beta-cells of the pancreatic islets, which are important regulator of blood glucose (Ma et al., 2010). Primarily, the loss or defect in insulin production or release from the beta-cell is caused by aberrant inflammatory response that results in a hyperglycemic state in the body of type 1 and 2 diabetes patients (Nauck, 2011). Multiple gene-based knockout studies and targeted protein knockdown approaches have established the importance of 12-LOX role in islet function. Insulin resistance and impairment in islet function that develops on a high-fat diet were prevented in leukocyte-12-LOX (12/15-LOX) knockout mice, suggesting that 12/15-LOX activity is relevant to type 2 diabetes, and to beta-cell dysfunction in obese states (Laybutt et al., 2002). Additionally, diabetic Zucker fatty rats that have a defect in insulin secretion have elevated 12-LOX expression/activity, further supporting a role for 12-LOX in the pathogenesis of type 2 diabetes (Tokuyama et al., 1995). A direct role of pro-inflammatory cytokines in stimulating 12-LOX activity is further supported by observations of cytokine-induced production of 12-HETE in both islets and beta-cell lines (Han et al., 2002). Furthermore, the addition of 12-LOX products (12-HETE and 12-HPETE) to human islets resulted in a decrease in glucose-stimulated insulin secretion associated with a decrease in islet viability (Persaud et al., 2007), and a partial restoration in glucose-stimulated insulin secretion if 12HETE was combined with lisofylline, an inhibitor of IL-12 signaling. Collectively these data support a predicted role of IL-12 in mediating the immune damage caused by the 12-LOX pathway (Simonsen et al., 1987; Vidgren et al., 1997; Vognild et al., 1998).

## CONCLUSION

Substantial gains have been made in our understanding of oxylipin signaling systems since their discovery 50 years ago. The recent advancements in mass spectrometry have led to a resurgence in oxylipin research. This technology has allowed for the accurate and reproducible measurement of over a 100 different oxylipins at the nanomolar level during cellular stimulation or complex signaling events such as blood coagulation (Strassburg et al., 2012). While these lipidomics techniques can identify the vast number of unique oxylipins produced during cellular stimulation, they do not address the biological function of the oxylipins generated. The level of resolution enabled by recent advances in lipidomics gives researchers the ability to discover novel oxylipins in an unbiased approach and measure oxylipin profiles from healthy individuals as well as those suffering from a variety of pathophysiological conditions.

The identification of novel oxylipins has outpaced the ability of researchers to characterize their biological functions. The characterization of individual oxylipin signaling is not a trivial matter. Further, multiple oxylipins are synthesized in response to the same agonist making it difficult to attribute the final cellular response to any one particular metabolite. Techniques to examine the role of individual oxylipins are not without their limitations, which is one of the reasons for much of the controversy in the field. Together a combination of lipidomics, genetics, and pharmacological approaches can approximate the role of an oxylipin in the cell and surrounding tissue. Finally, to identify the underlying mechanism regulated by an oxylipin of interest, its correlate receptor must be elucidated.

These new techniques can be used to identify potential biomarkers and therapeutic targets by identifying differences in the oxylipin profiles of healthy subjects and patients with a number of cardiovascular deficiencies including those with type 2 diabetes mellitus. This approach can be used to profile changes in the absolute magnitude of oxylipins or measure the ratios of oxylipins with juxtaposed signaling properties. One of the principle limitation to applying lipidomics techniques to identification of biomarkers and as a means to determine treatment is the availability of fresh samples. Thus, this technique is primarily being applied to the CVDs due to the ability to measure the oxylipin levels from serum and hematopoietic cells.

## Conflict of Interest Statement

The authors declare that the research was conducted in the absence of any commercial or financial relationships that could be construed as a potential conflict of interest.
